# Digital skin imaging applications, part I: Assessment of image acquisition technique features

**DOI:** 10.1111/srt.13163

**Published:** 2022-06-02

**Authors:** Mary D. Sun, Jonathan Kentley, Britney W. Wilson, H. Peter Soyer, Clara N. Curiel‐Lewandrowski, Veronica Rotemberg, Allan C. Halpern, ISIC Technique Working Group

**Affiliations:** ^1^ Icahn School of Medicine at Mount Sinai New York New York USA; ^2^ Dermatology Service Memorial Sloan Kettering New York New York USA; ^3^ Chelsea and Westminster Hospital London UK; ^4^ Rutgers New Jersey Medical School Newark New Jersey USA; ^5^ Dermatology Research Centre The University of Queensland Diamantina Institute Brisbane Queensland Australia; ^6^ Division of Dermatology University of Arizona Skin College of Medicine Tucson Arizona USA

**Keywords:** artificial intelligence, clinical imaging, digital tools, mobile applications, quality assurance, teledermatology

## Abstract

**Background:**

The rapid adoption of digital skin imaging applications has increased the utilization of smartphone‐acquired images in dermatology. While this has enormous potential for scaling the assessment of concerning skin lesions, the insufficient quality of many consumer/patient‐taken images can undermine clinical accuracy and potentially harm patients due to lack of diagnostic interpretability. We aim to characterize the current state of digital skin imaging applications and comprehensively assess how image acquisition features address image quality.

**Materials and methods:**

Publicly discoverable mobile, web, and desktop‐based skin imaging applications, identified through keyword searches in mobile app stores, Google Search queries, previous teledermatology studies, and expert recommendations were independently assessed by three reviewers. Applications were categorized by primary audience (consumer‐facing, nonhospital‐based practice, or enterprise/health system), function (education, store‐and‐forward teledermatology, live‐interactive teledermatology, electronic medical record adjunct/clinical imaging storage, or clinical triage), in‐app connection to a healthcare provider (yes or no), and user type (patient, provider, or both).

**Results:**

Just over half (57%) of 191 included skin imaging applications had at least one of 14 image acquisition technique features. Those that were consumer‐facing, intended for educational use, and designed for both patient and physician users had significantly greater feature richness (*p* < 0.05). The most common feature was the inclusion of text‐based imaging tips, followed by the requirement to submit multiple images and body area matching.

**Conclusion:**

Very few skin imaging applications included more than one image acquisition technique feature. Feature richness varied significantly by audience, function, and user categories. Users of digital dermatology tools should consider which applications have standardized features that improve image quality.

**Abbreviations**
AI: artificial intelligence
DTC: direct‐to‐consumer
EMR: electronic medical record
ISIC: International Skin Imaging Collaboration
NHB: non‐hospital‐based
S&F: store‐and‐forward
TD: teledermatology

## 1 INTRODUCTION

Digital health applications have become profoundly important in medicine. Although the adoption of these technologies was already growing prior to the COVID‐19 pandemic, recent restrictions have catalyzed a substantial and potentially permanent increase in US telehealth volumes.^1^, ^2^ The use of digital health tools for web and mobile‐based imaging are particularly useful in specialties that rely on visual assessment, including dermatology, plastic surgery, and oral‐maxillofacial surgery.^3^ Indeed, teledermatology (TD) is one of the most common uses of telemedicine, and skin conditions are consistently among the top five diagnoses associated with telehealth claims.^4^, ^5^ Global TD utilization has increased three‐fold due to the pandemic; patients and providers report high satisfaction and interest in future use.^4^, ^6^, ^7^

When implemented with rigorous standards and specialized equipment, there are high levels of agreement between TD and face‐to‐face management decisions as well as benefits to practice efficiencies and health equity.^7^, ^8^, ^9^, ^10^, ^11^, ^12^, ^13^, ^14^, ^15^, ^16^, ^17^, ^18^ However, an increasing number of TD services and other digital dermatology tools utilize “selfie” images taken by consumers/patients (discussed interchangeably), most commonly taken with smartphone cameras.^19^ While this has enormous potential for scaling the assessment of concerning skin lesions, the insufficient quality of many self‐acquired images can undermine the accuracy of tele‐triage and potentially harm patients. A recent systematic review of smartphone apps used for skin cancer detection found that nearly 20% of patient‐submitted images were not clinically interpretable.^20^ In apps that use underlying AI algorithms, image quality issues can lead to performance overestimation and impair categorization of lesion risk, especially in ulcerated, intertriginous, and large lesions.^21^, ^22^

In this study, we identify, categorize, and perform a comprehensive assessment of digital skin imaging applications for features addressing image acquisition technique. By including tools across multiple modalities and employing general dermatology search terms, we expand the scope of previous studies that have primarily focused on mobile apps and skin cancer detection.^21^, ^23^, ^24^, ^25^, ^26^, ^27^, ^28^, ^29^ We also synthesize an inventory of technique features, many of which address imaging guidelines previously proposed by International Skin Imaging Collaboration (ISIC),^30^, ^31^ that are currently implemented in digital skin imaging applications to improve image quality. This is the first feature‐level analysis of dermatology applications that assesses image quality improvement techniques.

## 2 METHODS

### 2.1 Search protocol and approach to identification of mobile apps

**FIGURE 1:**
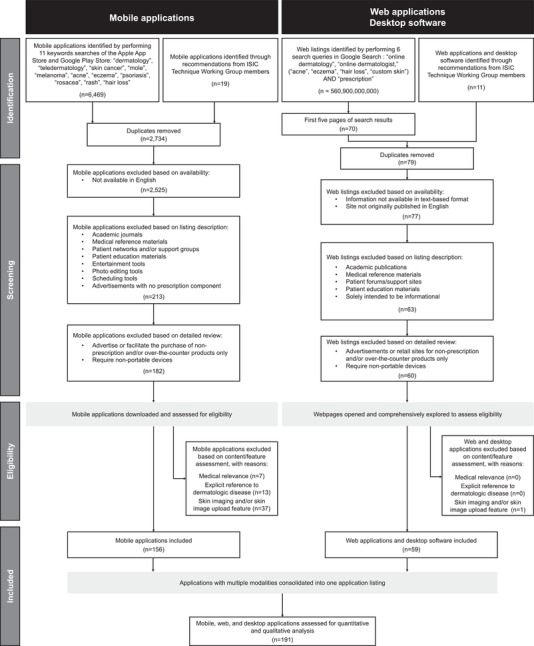
Flow diagram of search protocol used to identify mobile, web, and desktop skin imaging applications

The protocol for identifying mobile apps was designed in accordance with the *Quality and Risk of Bias Checklist for Studies that Review Smartphone Applications*.^32^ From January 1, 2021 through February 15, 2021, three reviewers conducted searches in the Apple App Store and Google Play Store, using three iPhone devices running iOS 14.4 and one Samsung Galaxy S10 device running Android 10, respectively. All devices were localized to the United States. Keywords used in previous dermatology app studies were used to query the Apple and Google app stores (Figure 1).^23^, ^25^, ^26^, ^33^ No date restrictions, language filters, or search filters were used.

MS, BW, and JK screened each mobile app based on the app listing properties, listing description, and listing screenshots. Remaining apps were downloaded and assessed for the following inclusion criteria: (1) medical relevance, (2) explicit references to dermatologic disease, and (3) the presence of skin imaging and/or skin image upload features. The first criterion was met if the app was categorized as Medical, or if the app listing included phrases associated with clinical action including but not limited to “treatment,” “prescription,” “doctor,” and “diagnosis.” The second criterion was met if dermatologic conditions were explicitly referenced prior to image upload. The third criterion was met if the app description included phrases such as “image upload,” “take a photo,” and “submit a picture,” if screenshots in the app listing showed these features, and/or based on manual exploration of the downloaded app. A detailed flowchart of the search protocol is provided in Figure 1.

### 2.2 Search protocol and approach to identification of web and desktop applications

From February 1, 2021 through February 15, 2021, the three reviewers conducted a parallel search for web‐ and desktop‐based skin imaging applications and software tools. Queries were run using the Google search engine, accessed from an incognito tab within the Google Chrome web browser. All devices were localized to the United State, and each reviewer's language setting in Chrome was English (United States) or English. Searches were run from the main search bar, and no additional search filters were used. Due to volume, narrower search terms were used than in the mobile app stores, and a limited number of results (the top 70 by Google web rank) were reviewed. Of note, *TD* was removed as a search term since many results were news articles or academic papers. Sponsored results were included, as they often linked to direct‐to‐consumer TD services and are highly visible to consumers. Listing descriptions and webpage content were evaluated for initial screening, after which inclusion and exclusion criteria were applied (Figure 1).

### 2.3 Identification of additional mobile, web, and desktop applications

A limited number of other skin imaging applications were considered based on academic studies of specific TD applications^21^, ^34^, ^35^, ^36^, ^37^, ^38^ and recommendations from members of the ISIC Technique Working Group (Figure 1). ISIC is an academic‐industry partnership that advances the application of digital skin imaging to reduce skin cancer mortality, formed by the International Society for Digital Imaging of the Skin. Recommenders from the Working Group were board‐certified dermatologists with specialized knowledge of commercial skin imaging. Eleven of the recommended applications were evaluated, and six were included.

### 2.4 Categorization and feature assessment of skin imaging applications

Each reviewer loaded the included mobile, web‐based, and desktop apps onto its corresponding smart device (iOS, Android, web browser, and/or desktop) and analyzed app characteristics and functionalities. The presence of paywalls or other restrictions was noted, and anonymous test accounts were made if needed to access features. For each recorded feature, an illustrative screenshot was taken for future reference. The application name, developer name, application modalities, availability, cost, user types (patient, provider, or both), and the availability of an in‐app connection to a licensed provider were also collected, if available.

Reviewers classified applications into one of three primary audiences (consumer‐facing, non‐hospital‐based [NHB] practices, or enterprise/health system) and one of five primary functions (educational, clinical triage, store‐and‐forward TD, live‐interactive TD, or electronic medical record [EMR] adjunct/clinical imaging storage), based on their content and main features. NHB practices were defined as solo and group practices. Apps were classified as primarily educational if skin images were not used for clinical diagnostic purposes, and/or if the app description explicitly stated that the app was educational (i.e., not intended as medical advice) even if a diagnosis was returned. Functionality was then assessed for any in‐app tools or features designed to improve image quality, resulting in an inventory of 14 image acquisition technique features (Table 1). Categorization and assessment were independently completed by each reviewer, and any discrepancies were resolved by consensus discussion.

**TABLE 1:** Definitions of 14 image acquisition technique features identified across included skin imaging applications

Category	#	Feature	Definition	Example
**User guidance**	1	Imaging tips	User‐facing text, of any length and location within the application, that provides recommendations or instructions for taking higher quality images	“Take your photo in a brightly lit area during the day.”
	2	Image area match	A focused area in the camera view that is intended to assist the user in centering their skin lesion	When the camera is opened within the app, the user sees a square overlay in the center of the viewfinder with everything outside of it slightly faded.
	3	Pose match	Any visual guide during the image capture process that provides guidance regarding how the user should pose or orient their body	*Mobile*: When the camera is opened within the app, the user sees a faded version of their last photo superimposed onto the viewfinder. *Web*: The user sees a cartoon figure posing.
	4	Lesion/border detection	The application automatically detects the borders of the lesion in the camera viewfinder	When getting ready to take a photo, the user sees a bright green outline of their lesion superimposed in the camera.
**Process of submission**	5	Multiple image requirement	Two or more images of the same skin complaint must be submitted for the primary outcome to occur	If multiple photos are not submitted, the user cannot proceed with booking their teledermatology session.
**Camera modifications**	6	Automated image capture	The in‐app camera captures an image without the user pressing on the camera button, usually after determining that conditions are sufficient	After the user moves to a brightly lit room, ensures that they are holding their smartphone still, and holds the camera very close to their skin, an image is automatically taken and uploaded to the application.
	7	Adjusted camera function	Additional features that the in‐app camera has outside the normal capabilities of the default smartphone camera app	The user can move where the image capture (camera) button is on their phone screen so that it is most convenient for them to use when imaging their skin.
	8	Automated camera settings	Automatic application of specific camera settings to the in‐app image capture process	When the in‐app camera is launched, phone brightness automatically increases and flash is turned on.
**Automated feedback**	9	Auto‐rejection and/or suggested retry	Upon the submission of images determined to be insufficient, new images are requested, and/or the application flow cannot proceed until new photos are uploaded and determined to be sufficient	After uploading a photo of their pet, the user sees an in‐camera message: “This does not appear to be a photo of skin. Please upload a more accurate photo to proceed.”
	10	Light balance detection	The application automatically detects if there is insufficient lighting for the image and alerts the user	As the user is getting ready to take a photo in a dark room, they see an in‐camera message: “Move to a brighter area.”
	11	Blur/focus detection	The application automatically detects if the lesion is blurry or not in focus and alerts the user	After the user takes a blurry image of their skin lesion, they see an in‐camera message: “This picture is not in focus—try again!”
	12	Distance detection	The application automatically detects if the lesion is too far away from the camera and alerts the user	When holding their camera 10 inches away from their arm to take a photo, the user sees an in‐camera message: “Too far away, move your camera closer.”
	13	Obscuration detection	The application automatically detects if the lesion is obscured and alerts the user	As the user is getting ready to take a photo of a lesion partially covered by their bracelet, they see an in‐camera message: “Please make sure your skin is uncovered.”
	14	Camera angle detection	The application automatically detects if the camera angle is not close to perpendicular and alerts the user	When holding their camera tilted forward at a 45‐degree angle to take a photo, the user sees an in‐camera message: “Please hold your phone flat over your skin.”

### 2.5 Statistical methods

Two‐way ANOVA was conducted to determine if the mean number of technique features per skin imaging application differed significantly across categories (*p* < 0.05). All analyses and figures were generated in R statistical programming software, version 4.02.^39^

## 3 RESULTS

A total of 191 mobile, web, and desktop skin imaging applications were included in this study, nearly all (96%) of which were publicly discoverable. The majority (68%) of skin imaging applications was consumer‐facing, 12% were intended for patients of NHB practices, and 21% were targeted to enterprise customers and/or health systems. Approximately one third (34%) of apps were designed solely for patient use, 14% for providers, and over half (52%) accommodated both. The latter were usually store‐and‐forward TD services, where patients upload photos, provide additional information regarding cutaneous complaints, and submit payment prior to being connected with clinicians. Store‐and‐forward TD tools accounted for 50% of all skin imaging applications, followed by education apps at 33%, EMR adjuncts/clinical imaging tools at 8%, live‐interactive TD at 7%, and clinical triage at only 1%. Notably, 63% of apps facilitated an in‐app connection to a licensed healthcare provider (Table S1).

**FIGURE 2:**
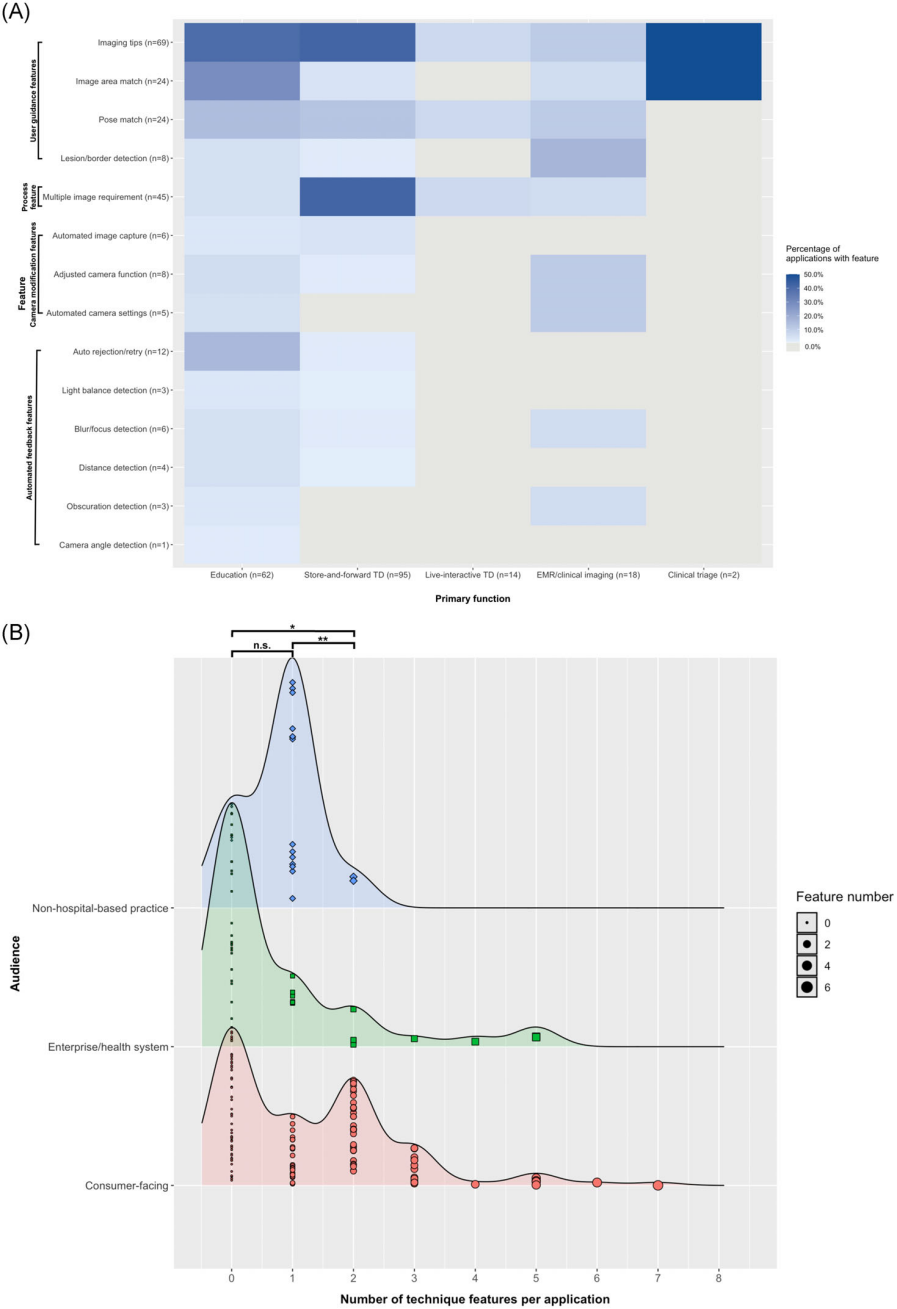
Image acquisition technique feature density across skin imaging applications audience and primary function. (A) Density plot of the number of technique features per application within each primary audience type. The size of each jittered data point increases with the number of features per application. Significance bars at the top of the plot indicate if statistically significant differences were found between the mean number of technique features per application across audience categories (***p < 0.001, **p < 0.01, *p < 0.05, n.s. = *p* > 0.05). (B) Heatmap of the frequency of specific technique features across all applications, relative to primary function. Features are grouped and labeled by functional category on the y‐axis. Darker blue hues correspond to higher percentages, and gray indicates 0%

Just over half (57%) of all skin imaging applications included any image acquisition technique features, with an average of 1.1 features per application (Table 2). Nearly two thirds (66%) of applications had only one or no technique features, followed by 29% with two to three features, and 5% with four to six features (Table 2). Feature richness varied significantly by audience, primary function, and user type, where applications categorized as consumer‐facing, educational, and intended for both patient and physician users had more features, on average (Table 2; Figure 2A). Most applications with only one feature, which was most commonly the requirement to submit multiple images (“multiple image requirement”), were associated with NHB dermatology practices (Table 2). Applications with the greatest number of technique features overall, including My Acnie Acne, SkinVision, SkinIO, and MoleScope, were developed for either consumer or enterprise/health system audiences (Figure 2A; Table 3).

**TABLE 2:** Number of image acquisition technique features per application, by application characteristic

	0–1 features	2–3 features	4–6 features	Category total	*p*‐Value
**Audience (*n* = 191)**					
Consumer‐facing	74 (57%)	48 (37%)	7 (5%)	129 (100%)	0.041^*^
Nonhospital‐based practice	20 (91%)	2 (9%)	0 (0%)	22 (100%)	
Enterprise/health system	32 (80%)	5 (13%)	3 (8%)	40 (100%)	
**Function (*n* = 191)**					
Education	36 (57%)	23 (37%)	4 (6%)	63 (100%)	0.038^*^
Store‐and‐forward TD	62 (65%)	28 (30%)	5 (5%)	95 (100%)	
Live‐interactive TD	13 (93%)	1 (7%)	0 (0.0%)	14 (100%)	
Clinical triage	1 (50%)	1 (50%)	0 (0.0%)	2 (100%)	
EMR/clinical imaging	14 (78%)	3 (17%)	1 (6%)	18 (100%)	
**In‐app connection to provider (*n* = 191)**					
Yes	83 (69%)	30 (25%)	8 (8%)	121 (100%)	0.817
No	43 (61%)	25 (36%)	2 (3%)	70 (100%)	
**User type (*n* = 191)**					
Patient only	40 (63%)	21 (33%)	3 (5%)	64 (100%)	0.036*
Provider only	23 (85%)	4 (15%)	0 (0%)	27 (100%)	
Both	63 (63%)	30 (30%)	7 (7%)	100 (100%)	

Abbreviations: EMR, electronic medical record; TD, teledermatology.

**TABLE 3:** Image acquisition technique features identified in the top five skin imaging applications by technique feature count, per audience category

			User guidance				Process	Camera modifications			Automated feedback					
#	App name	Primary function	Imaging tips	Image area match	Pose match	Lesion/border detection	Multiple image rqmt.	Auto image capture	Adjusted camera function	Auto camera settings	Auto reject/retry	Light detect	Blur detect	Distance detect	Obscuration detect	Camera angle detect
**Consumer‐facing**																
1	My Acnie Acne	Education	**✓**		**✓**		**✓**	**✓**			**✓**			**✓**		**✓**
2	SkinVision	Education				**✓**					**✓**	**✓**	**✓**	**✓**	**✓**	
3	MoleScope	S&F TD	**✓**		**✓**		**✓**	**✓**	**✓**							
4	MiiSkin	Education	**✓**		**✓**	**✓**		**✓**						**✓**		
5	Rash ID	Education	**✓**								**✓**	**✓**	**✓**		**✓**	
**Nonhospital‐based practice** *																
1	DMC Telederm	S&D TD	**✓**				**✓**									
2	OneSkin Derm.	S&F TD	**✓**				**✓**									
**Enterprise/Health system**																
1	SkinIO	S&F TD	**✓**		**✓**	**✓**			**✓**				**✓**			
2	MatchLab AI	S&F TD	**✓**		**✓**			**✓**				**✓**	**✓**			
3	Derm‐Engine	EMR/Clinical imaging	**✓**		**✓**	**✓**									**✓**	
4	HEINE NC2	EMR/Clinical imaging					**✓**		**✓**	**✓**						
5	Epitomyze Capture	EMR/Clinical imaging	**✓**		**✓**											

Abbreviations: EMR, electronic medical record; S&F, store‐and‐forward; TD, teledermatology.

* Only two applications in the nonhospital‐based practice category had more than one technique feature.

Across all applications, the most commonly occurring features were those designed to provide user guidance during imaging and to optimize the process of image submission. In order of frequency, these included imaging tips, the requirement to submit multiple images, pose match, image area match, and auto rejection/retry (Figure 2B). Applications targeted to consumers and enterprise companies/health systems demonstrated the widest range of features, compared to those developed by NHB practices. In consumer‐facing applications, imaging tips, the requirement to submit multiple images, image area match, and pose match were the most commonly occurring features, while imaging tips, pose match, and lesion/border detection features occurred most often in enterprise/health system applications. Imaging tips and the requirement to submit multiple images were the only features that occurred in applications developed by NHB practices (Table S1). Among the top five applications by feature count in each audience category, consumer‐facing and enterprise tools had substantially more feature diversity when compared to applications associated with NHB practices. These applications primarily implemented features that provided user guidance, augmented camera functions, and generated automated user feedback during the imaging process (Table 3).

When considering applications by primary function, we found that education, store‐and‐forward TD, and EMR adjunct/clinical imaging tools demonstrated the greatest feature diversity with 14 (100%), 11 (79%), and 9 (62%) of technique features represented, respectively (Figure 2B). Many technique features were most highly represented in educational skin imaging tools, with the exception of imaging tips and the requirement to submit multiple images. These two features were most common within (Figure 2B) and indeed co‐occurred in 42% of store‐and‐forward TD applications. Image area match and auto rejection/suggested retry features were more frequent in applications intended for educational use compared to other primary functions, while pose match was relatively similarly represented across education, store‐and‐forward TD, and EMR/clinical imaging applications. Among EMR adjunct/clinical imaging tools, adjusted camera function and automated camera settings features, as well as lesion/border detection features occurred at relatively high percentages (Figure 2B). While imaging tips and image area match occurred at 50% frequency in clinical triage apps, only two applications were classified within this category, and only one demonstrated any technique features.

## 4 DISCUSSION

Our findings show that there is a strong need for skin imaging applications to implement more robust functionality that addresses image acquisition technique and quality assurance. Of the 191 publicly discoverable skin imaging tools, we identified across mobile, web, and desktop modalities, 66% had one or fewer technique‐related imaging features (Table 2). Imaging tips, which were the most common feature, varied significantly in quality and discoverability. For example, instructional content ranged from a few general phrases to precise, illustrated, and well‐annotated visual guides and videos. Although these tips were usually integrated as part of the imaging workflow in mobile apps, many web‐based applications included them only in separate frequently asked questions or product information pages. The second most common feature was the requirement to submit multiple images, which can improve the chance of submitting a clinically sufficient photo. However, the usefulness of submitting multiple images is limited by the quality of those images. This requirement was often the only technique‐related feature identified in store‐and‐forward apps, despite clear synergies with other technique features. Overall, the existing level of technique features in digital applications is low, and even common features are not standardized.

Emphasizing image quality standards in skin imaging tools is particularly important, as many of these applications are publicly discoverable, consumer‐facing, and largely (83%) used for educational and store‐and‐forward TD purposes. Although the inclusion of technique features was limited overall, applications in these categories also exhibited a more varied set of features and were more feature‐rich, on average. While we find the inclusion of additional technique features in consumer‐facing apps to be intuitive, as laypersons may be less likely to take high‐quality pictures without specific instruction, we were surprised to see higher feature numbers in educational apps. We determined that a substantial portion of education‐classified applications are actually AI diagnostic tools that provide automated assessments of skin lesion risk. To avoid establishing a patient‐physician relationship, many of these applications include disclaimers stating that diagnoses are intended for educational purposes. Concerningly, we were frequently able to submit and receive “diagnoses” for images that were clearly not of skin (e.g., photos of a houseplant), with no feedback or warning. These exploratory findings are consistent with previous studies that find poor performance in some algorithm‐based smartphone apps and underscore the need for quality assurance with regard to image quality.^21^, ^40^, ^41^ While “educational” AI applications may not be directly involved in clinical encounters, their outputs can affect health‐seeking behaviors and cause patient distress.

Similarly, achieving high image quality is important when capturing images that will be used directly in clinical settings. Skin imaging applications that capture photos used for clinical triage, TD services, and as EMR adjuncts/clinical image storage should be more likely to implement technique features that ensure high quality images, especially since these images become part of the medical record and play a role in clinical management. However, these expectations were not reflected in our findings. While the proportion of store‐and‐forward TD tools with at least one technique feature (42%) was the highest relative to other function categories, this still seems relatively low given the centrality of patient‐uploaded images to clinical decision‐making in store‐and‐forward TD. This percentage was only 8% in EMR adjunct/clinical imaging applications and 6% in live‐interactive TD applications; image upload functionality may be less important in the setting of live‐interactive TD.

Consumer‐facing tools had the largest proportion of applications with more than two technique features, while those developed by NHB practices had the lowest proportions. This is particularly concerning since 96% of the latter are store‐and‐forward TD tools. Interestingly, the relative proportion of patient‐targeted applications with at least two technique features was four times that of provider‐targeted applications. This may reflect the assumption that patients benefit more from technique features than clinicians do, even though medical professionals often operate in time‐poor settings and can derive significant value from built‐in quality assurance processes. Few resources are designed to educate patients or providers about mobile image capture, although the CLOSE‐UP imaging guide was recently proposed for clinicians.^42^ This dearth of image capture training contributes to common findings of blurriness, poor lighting, and other environmental issues in images utilized for TD, even in those acquired during medical visits.^42^, ^43^ Developers of skin imaging applications should prioritize the inclusion of technique features to improve image quality, regardless of user type.

There were several limitations in our study. First, our ability to access all imaging functionality was limited by built‐in paywalls (full paywalls prohibited the review of 10% of included apps), in‐app purchases, paid features, and paid memberships (found in 68% of included apps), and closed beta programs (used in 4% of included apps). Some of the companies we included may also implement customized versions of software for different clients and may include backend quality review processes. Our analysis may therefore underreport features not included in free product versions and be biased toward applications that are publicly discoverable and free to use. Second, we did not include major EMR companies (e.g., Epic) due to lack of specificity for skin disease and barriers to access. In practice, many dermatologists likely use these tools for image capture. Third, initial searches were conducted using devices localized to the United States and excluded applications not available in English. Therefore, our study does not capture the full range of publicly discoverable skin imaging apps that may be used in other countries. Additionally, we acknowledge that reporting feature counts only treats all features as though they are of equal value.

## 5 CONCLUSIONS

Our findings reveal the existing dearth of technique features and quality assurance functionality across a wide variety of publicly discoverable, mostly consumer‐facing mobile, web‐based, and desktop skin imaging applications. Although recommendations for image acquisition exist in the dermatology literature,^30^, ^31^, ^42^, ^44^ existing instruction across digital applications is highly heterogenous and nonstandardized. The implementation of universal imaging standards, which do not yet exist in dermatology, could provide valuable guidance to app developers, establish standards for skin imaging devices, and standardize features that benefit clinicians and patients alike. AI‐based diagnostic tools, despite their nonclinical designations, should implement additional quality safeguards and be more thoroughly monitored for quality.^41^ Educational resources should also be developed to assist consumers with mobile image capture, especially in TD workflows.

Despite the obvious impact of image quality on the utility of digital images in dermatology, there has been limited attention paid to imaging techniques across skin imaging applications. Dermatology tools that utilize user‐uploaded photos should incorporate image quality techniques informed by further research regarding the efficacy of various approaches. Future evaluations of digital skin imaging tools, across all audience and function categories, can broaden the scope of analysis to include other clinically relevant features and technology standards that affect interoperability. To avoid patient harm and fully realize the benefits of telehealth, improving the clinical viability of digital dermatology images must be prioritized.

## CONFLICT OF INTEREST

H. Peter Soyer is a shareholder of MoleMap NZ Limited and e‐derm consult GmbH and undertakes regular teledermatological reporting for both companies. H. Peter Soyer is a Medical Consultant for Canfield Scientific Inc., MoleMap Australia Pty Limited, a Medical Advisor for First Derm, and Revenio Research Oy. Allan C. Halpern has equity interests in HCW Health, LLC and owns intellectual property rights in SKIP Derm, LLC. Allan C. Halpern is a Medical Consultant for Canfield Scientific Inc., Lloyd Charitable Trust, and SciBASE. Other authors have no conflict of interest to disclose.

## FUNDING INFORMATION

None.

## Supporting information

Supporting information.

## Data Availability

The data that supports the findings of this study are available in the supplementary material of this article.
