# Supplementary material for: Digital skin imaging applications, part I: Assessment of image acquisition technique features
Source: Skin Res Technol. 2022 Jun 2;28(4):623–32. doi: 10.1111/srt.13163 (PMC9907654; doi:10.1111/srt.13163)
Supplement: Supplementary file 1 — Supporting information. [file SRT-28-623-s001.docx]

Supplementary Table 1. Characteristics and feature inventory of digital skin imaging applications with ≥1 image acquisition technique feature, per audience category (n=108)

| **#** | **Name** | **Modality** | **Primary Function** | **User^*^** | **Connect to provider** | **User guidance**  **features** | | | | **Process feature** | **Camera**  **features** | | | **Automated feedback**  **features** | | | | | |
| --- | --- | --- | --- | --- | --- | --- | --- | --- | --- | --- | --- | --- | --- | --- | --- | --- | --- | --- | --- |
|  |  |  |  |  |  | Imaging tips | Image area match | Pose match | Lesion/  border detection | Multiple image rqmt. | Auto image capture | Adjusted camera function | Auto camera settings | Auto reject/  retry | Light detect | Blur detect | Distance detect | Obscur-ation detect | Camera angle detect |
| **CONSUMER-FACING** | | | | | | | | | | | | | | | | | | | |
| 1 | My Acnie Acne progress tracker | iOS | Education | PT |  | **✓** |  | **✓** |  | **✓** | **✓** |  |  | **✓** |  |  | **✓** |  | **✓** |
| 2 | SkinVision | iOS, Android | Education | PT, PR | **✓** |  |  |  | **✓** |  |  |  |  | **✓** | **✓** | **✓** | **✓** | **✓** |  |
| 3 | MoleScope | iOS, Android, Device | S&F TD | PT | **✓** | **✓** |  | **✓** |  | **✓** | **✓** | **✓** |  |  |  |  |  |  |  |
| 4 | MiiSkin Skin Tracker/PRO | iOs, Android, Web | Education | PT, PR | **✓** | **✓** |  | **✓** | **✓** |  | **✓** |  |  |  |  |  | **✓** |  |  |
| 5 | MDacne | iOS, Android | S&F TD | PT, PR | **✓** | **✓** |  | **✓** | **✓** |  | **✓** |  |  |  |  |  | **✓** |  |  |
| 6 | Rash ID | iOS | Education | PT |  | **✓** |  |  |  |  |  |  |  | **✓** | **✓** | **✓** |  | **✓** |  |
| 7 | Hims | Web | S&F TD | PT, PR | **✓** | **✓** |  | **✓** |  | **✓** |  |  |  | **✓** |  |  |  |  |  |
| 8 | Imagine - Skin Condition Tracker | iOs, Android, Web | Education | PT |  | **✓** | **✓** | **✓** |  |  |  |  |  |  |  |  |  |  |  |
| 9 | Medgic - Scan, Analyze and Detect Skin Problems | Android | Education | PT |  | **✓** | **✓** |  | **✓** |  |  |  |  |  |  |  |  |  |  |
| 10 | AI Dermatologist: Skin Scanner | iOS | Education |  |  | **✓** | **✓** |  |  |  |  |  |  | **✓** |  |  |  |  |  |
| 11 | Hers | Web | S&F TD | PT, PR | **✓** | **✓** |  | **✓** |  | **✓** |  |  |  |  |  |  |  |  |  |
| 12 | Software | Web | S&F TD | PT, PR | **✓** | **✓** |  | **✓** |  | **✓** |  |  |  |  |  |  |  |  |  |
| 13 | HealthAI | iOS | Education | PT | **✓** | **✓** |  |  |  |  |  |  |  | **✓** |  | **✓** |  |  |  |
| 14 | Klear Acne | iOS, Android | S&F TD | PT, PR | **✓** | **✓** |  | **✓** |  | **✓** |  |  |  |  |  |  |  |  |  |
| 15 | Roman | Web | S&F TD | PT, PR | **✓** | **✓** |  | **✓** |  |  |  |  |  | **✓** |  |  |  |  |  |
| 16 | ClearifiRx | Web | S&F TD | PT, PR | **✓** | **✓** |  | **✓** |  | **✓** |  |  |  |  |  |  |  |  |  |
| 17 | iDoc24 | iOS, Android | S&F TD | PT, PR | **✓** | **✓** | **✓** |  |  | **✓** |  |  |  |  |  |  |  |  |  |
| 18 | STD Triage Ask dermatology | iOS, Android | S&F TD | PT, PR | **✓** | **✓** | **✓** |  |  | **✓** |  |  |  |  |  |  |  |  |  |
| 19 | QSkin | Android | Education | PT |  | **✓** | **✓** |  |  |  |  | **✓** |  |  |  |  |  |  |  |
| 20 | Lemonaid | iOS, Android, Web | Live TD | PT, PR | **✓** | **✓** |  | **✓** |  | **✓** |  |  |  |  |  |  |  |  |  |
| 21 | CompariSkin Basic | iOS | Education | PT |  | **✓** |  | **✓** |  |  |  |  |  |  |  |  |  |  |  |
| 22 | CompariSkin | iOS | Education | PT |  | **✓** |  | **✓** |  |  |  |  |  |  |  |  |  |  |  |
| 23 | Triage | Web | Education | PT, PR |  | **✓** |  |  |  |  |  |  |  | **✓** |  |  |  |  |  |
| 24 | Molexplore | iOS, Android | Education | PT |  |  | **✓** | **✓** |  |  |  |  |  |  |  |  |  |  |  |
| 25 | Mole Mapper Melanoma Study | iOS | Education | PT |  | **✓** | **✓** |  |  |  |  |  |  |  |  |  |  |  |  |
| 26 | MyForte | iOS | Education | PT |  | **✓** |  | **✓** |  |  |  |  |  |  |  |  |  |  |  |
| 27 | Acne Diary | iOS | Education |  |  |  |  | **✓** |  | **✓** |  |  |  |  |  |  |  |  |  |
| 28 | Bitskin - Skin cancer scanner and tracker | Android | Education | PT |  |  | **✓** |  |  |  |  | **✓** |  |  |  |  |  |  |  |
| 29 | Skin Cancer Risk Prediction | Android | Education | PT |  |  | **✓** |  |  |  |  | **✓** |  |  |  |  |  |  |  |
| 30 | Skin Monitor: check your moles | Android | Education | PT |  | **✓** |  |  |  |  |  | **✓** |  |  |  |  |  |  |  |
| 31 | Curology | Web | S&F TD | PT, PR | **✓** | **✓** |  |  |  | **✓** |  |  |  |  |  |  |  |  |  |
| 32 | Rash Decision | Android | Education | PT |  | **✓** | **✓** |  |  |  |  |  |  |  |  |  |  |  |  |
| 33 | My Skin App | iOS | Education | PT |  | **✓** | **✓** |  |  |  |  |  |  |  |  |  |  |  |  |
| 34 | DermMeasure | iOS | Education | PT, PR |  | **✓** | **✓** |  |  |  |  |  |  |  |  |  |  |  |  |
| 35 | SkinScreen | Android | Education | PT |  |  | **✓** |  |  |  |  |  |  | **✓** |  |  |  |  |  |
| 36 | Deep Learning for Melanoma | Android | Education | PT |  | **✓** | **✓** |  |  |  |  |  |  |  |  |  |  |  |  |
| 37 | Keeps | Web | S&F TD | PT, PR | **✓** | **✓** |  | **✓** |  |  |  |  |  |  |  |  |  |  |  |
| 38 | Dermatica | Web | S&F TD | PT, PR | **✓** | **✓** |  |  |  | **✓** |  |  |  |  |  |  |  |  |  |
| 39 | Rory | Web | S&F TD | PT, PR | **✓** | **✓** |  | **✓** |  |  |  |  |  |  |  |  |  |  |  |
| 40 | Strut | Web | S&F TD | PT, PR | **✓** | **✓** |  |  |  | **✓** |  |  |  |  |  |  |  |  |  |
| 41 | DearBrightly | Web | S&F TD | PT, PR | **✓** | **✓** |  |  |  | **✓** |  |  |  |  |  |  |  |  |  |
| 42 | DirectDerm | Web | S&F TD | PT, PR | **✓** | **✓** |  |  |  | **✓** |  |  |  |  |  |  |  |  |  |
| 43 | Maple | Web | S&F TD | PT, PR | **✓** | **✓** |  |  |  | **✓** |  |  |  |  |  |  |  |  |  |
| 44 | First Derm Online Dermatology | iOS, Android, Web | S&F TD | PT, PR | **✓** | **✓** | **✓** |  |  |  |  |  |  |  |  |  |  |  |  |
| 45 | SkyMD | iOS, Android, Web | S&F TD | PT, PR | **✓** | **✓** |  |  |  | **✓** |  |  |  |  |  |  |  |  |  |
| 46 | scanoma - mole check | iOS, Android | S&F TD | PT, PR | **✓** | **✓** | **✓** |  |  |  |  |  |  |  |  |  |  |  |  |
| 47 | Teladoc | iOS, Android, Web | S&F TD | PT, PR | **✓** | **✓** |  |  |  | **✓** |  |  |  |  |  |  |  |  |  |
| 48 | Qr8 MediSkin | Web | S&F TD | PT, PR | **✓** | **✓** |  |  |  | **✓** |  |  |  |  |  |  |  |  |  |
| 49 | Trulee | Web | S&F TD | PT, PR | **✓** | **✓** |  |  |  | **✓** |  |  |  |  |  |  |  |  |  |
| 50 | Skin+Me | Web | S&F TD | PT, PR | **✓** | **✓** |  |  |  | **✓** |  |  |  |  |  |  |  |  |  |
| 51 | SnapMed | Web | S&F TD | PT, PR | **✓** | **✓** |  |  |  | **✓** |  |  |  |  |  |  |  |  |  |
| 52 | Virtuwell | Web | S&F TD | PT, PR | **✓** | **✓** |  |  |  | **✓** |  |  |  |  |  |  |  |  |  |
| 53 | Skin Cancer Detector | iOS | Education | PT |  | **✓** | **✓** |  |  |  |  |  |  |  |  |  |  |  |  |
| 54 | Model Dermatology | Android | Education | PT |  | **✓** |  |  |  | **✓** |  |  |  |  |  |  |  |  |  |
| 55 | dermaGO | Web | S&F TD | PT, PR | **✓** | **✓** |  |  |  | **✓** |  |  |  |  |  |  |  |  |  |
| 56 | DermIA Pro - Analyze Skin Cancer with your camera | Android | Education | PT |  | **✓** |  |  |  |  |  |  |  |  |  |  |  |  |  |
| 57 | MySkinSelfie | iOS, Android, Web | Education | PT, PR |  |  |  | **✓** |  |  |  |  |  |  |  |  |  |  |  |
| 58 | Track Your Spot | iOS | Education | PT |  |  | **✓** |  |  |  |  |  |  |  |  |  |  |  |  |
| 59 | Psoriasis Monitor | iOS, Android | Education | PT | **✓** |  | **✓** |  |  |  |  |  |  |  |  |  |  |  |  |
| 60 | Smart Skin Cancer Detection | Android | Education | PT |  |  |  |  |  |  |  |  | **✓** |  |  |  |  |  |  |
| 61 | Skin Cancer Check | iOS | Education | PT |  |  | **✓** |  |  |  |  |  |  |  |  |  |  |  |  |
| 62 | EczemaLess, an AI Eczema Guide; AI Eczema App: A tool to manage Eczema | iOS, Android | Education | PT |  |  |  |  |  |  |  |  |  | **✓** |  |  |  |  |  |
| 63 | Beat-Psoriasis: AI management; AI Psoriasis App: Manage and Care | iOS, Android | Education | PT | **✓** |  |  |  |  |  |  |  |  | **✓** |  |  |  |  |  |
| 64 | DermIA - Analyze Skin Cancer with your camera A.I | Android | Education | PT |  | **✓** |  |  |  |  |  |  |  |  |  |  |  |  |  |
| 65 | MoleAgnose | Android | Education | PT |  | **✓** |  |  |  |  |  |  |  |  |  |  |  |  |  |
| 66 | Snap Skin | Android | S&F TD | PT | **✓** | **✓** |  |  |  |  |  |  |  |  |  |  |  |  |  |
| 67 | UMSkinCheck | iOS, Android | Education | PT |  | **✓** |  |  |  |  |  |  |  |  |  |  |  |  |  |
| 68 | Aysa | iOS, Android | Education | PT |  |  | **✓** |  |  |  |  |  |  |  |  |  |  |  |  |
| 69 | SKLIP | iOS, Android | S&F TD | PT, PT | **✓** | **✓** |  |  |  |  |  |  |  |  |  |  |  |  |  |
| 70 | Skin Doctor | iOS, Android | Education | PT |  |  |  |  |  |  |  |  | **✓** |  |  |  |  |  |  |
| 71 | DermatologistOnCall | Android, Web | S&F TD | PT, PR | **✓** |  |  |  |  | **✓** |  |  |  |  |  |  |  |  |  |
| 72 | Visus: Skin Cancer Detection | Android | Education | PT |  |  |  |  |  |  |  |  | **✓** |  |  |  |  |  |  |
| 73 | DermaPhoto by Skinzy: Skin Disease Prediction | Android | Education | PT, PR | **✓** |  |  |  |  |  |  |  |  | **✓** |  |  |  |  |  |
| 74 | DermoScanner | Android | Education | PT |  | **✓** |  |  |  |  |  |  |  |  |  |  |  |  |  |
| 75 | CallonDoc | Web | S&F TD | PT, PR | **✓** |  |  |  |  | **✓** |  |  |  |  |  |  |  |  |  |
| 76 | DocBright | Web | S&F TD | PT, PR | **✓** | **✓** |  |  |  |  |  |  |  |  |  |  |  |  |  |
| 77 | DermCafé Canada | Web | S&F TD | PT, PR | **✓** | **✓** |  |  |  |  |  |  |  |  |  |  |  |  |  |
| **NON-HOSPITAL-BASED PRACTICE** | | | | | | | | | | | | | | | | | | | |
| 78 | DMC Telederm | iOS, Android | S&F TD | PT, PR | **✓** | **✓** |  |  |  | **✓** |  |  |  |  |  |  |  |  |  |
| 79 | OneSkin Dermatology | Web | S&F TD | PT | **✓** | **✓** |  |  |  | **✓** |  |  |  |  |  |  |  |  |  |
| 80 | Forefront Dermatology | Android | S&F TD | PT, PR | **✓** |  |  |  |  | **✓** |  |  |  |  |  |  |  |  |  |
| 81 | Advanced Dermatology (Derm Care) | Android | S&F TD | PT, PR | **✓** |  |  |  |  | **✓** |  |  |  |  |  |  |  |  |  |
| 82 | Dermatology Specialists | Android | S&F TD | PT, PR | **✓** |  |  |  |  | **✓** |  |  |  |  |  |  |  |  |  |
| 83 | Bella Pelle Dermatology | Android | S&F TD | PT, PR | **✓** |  |  |  |  | **✓** |  |  |  |  |  |  |  |  |  |
| 84 | Summa Health Dermatology | Android, Web | S&F TD | PT, PR | **✓** |  |  |  |  | **✓** |  |  |  |  |  |  |  |  |  |
| 85 | Advanced Dermatology SCC | Android | S&F TD | PT, PR | **✓** |  |  |  |  | **✓** |  |  |  |  |  |  |  |  |  |
| 86 | Associates in Dermatology | Android | S&F TD | PT, PR | **✓** |  |  |  |  | **✓** |  |  |  |  |  |  |  |  |  |
| 87 | Dermatology Consultants | Android, Web | S&F TD | PT, PR | **✓** |  |  |  |  | **✓** |  |  |  |  |  |  |  |  |  |
| 88 | Arnot Health Dermatology | Android | S&F TD | PT, PR | **✓** |  |  |  |  | **✓** |  |  |  |  |  |  |  |  |  |
| 89 | Pine Belt Dermatology | Android | S&F TD | PT, PR | **✓** |  |  |  |  | **✓** |  |  |  |  |  |  |  |  |  |
| 90 | United Skin Specialists | Android | S&F TD | PT, PR | **✓** |  |  |  |  | **✓** |  |  |  |  |  |  |  |  |  |
| 91 | AHN DermOnCall | Android | S&F TD | PT, PR | **✓** |  |  |  |  | **✓** |  |  |  |  |  |  |  |  |  |
| 92 | BHSkin | Android | S&F TD | PT, PR | **✓** |  |  |  |  | **✓** |  |  |  |  |  |  |  |  |  |
| 93 | Online Skin Specialist | Web | S&F TD | PT, PR | **✓** | **✓** |  |  |  |  |  |  |  |  |  |  |  |  |  |
| **ENTERPRISE/HEALTH SYSTEM** | | | | | | | | | | | | | | | | | | | |
| 94 | MatchLab AI | iOS, Android | S&F TD | PT, PR | **✓** | **✓** |  | **✓** |  |  | **✓** |  |  |  | **✓** | **✓** |  |  |  |
| 95 | SkinIO | iOS, Web | S&F TD | PT, PR | **✓** | **✓** |  | **✓** | **✓** |  |  | **✓** |  |  |  | **✓** |  |  |  |
| 96 | DermEngine | iOS, Android, Web, Desktop | EMR/  Clinical imaging | PT, PR | **✓** | **✓** |  | **✓** | **✓** |  |  |  |  |  |  |  |  | **✓** |  |
| 97 | HEINE NC2 | iOS, Device | EMR/  Clinical imaging | PR |  |  |  |  |  | **✓** |  | **✓** | **✓** |  |  |  |  |  |  |
| 98 | Imagine for Studies | iOS, Android | S&F TD | PT, PR | **✓** | **✓** |  |  |  |  | **✓** |  |  |  |  |  |  |  |  |
| 99 | Epitomyze Capture | iOS | EMR/  Clinical imaging | PR |  | **✓** |  | **✓** |  |  |  |  |  |  |  |  |  |  |  |
| 100 | DermaAId | Android | Clinical Triage | PR | **✓** | **✓** | **✓** |  |  |  |  |  |  |  |  |  |  |  |  |
| 101 | M.E.D.X. | iOS, Android | EMR/  Clinical imaging | PR, PT |  |  | **✓** |  |  |  |  | **✓** |  |  |  |  |  |  |  |
| 102 | FotoFinder Hub | Web, Device | EMR/  Clinical imaging | PR | **✓** |  |  |  | **✓** |  |  |  |  |  |  |  |  |  |  |
| 103 | SCARLETRED System | iOS, Desktop, Device | S&F TD | PR | **✓** | **✓** |  |  |  |  |  |  |  |  |  |  |  |  |  |
| 104 | HEINE DERM | iOS, Android, Device | EMR/  Clinical imaging | PR |  |  |  |  |  |  |  |  | **✓** |  |  |  |  |  |  |
| 105 | imitoCam Enterprise | iOS, Android | EMR/  Clinical imaging | PR |  |  |  |  | **✓** |  |  |  |  |  |  |  |  |  |  |
| 106 | handyscope pro | iOS, Android, Device | EMR/  Clinical imaging | PR | **✓** |  |  |  |  |  |  |  |  |  |  | **✓** |  |  |  |
| 107 | ClickMedix (ClickDerm) | iOS | S&F TD | PT, PR | **✓** |  |  |  |  | **✓** |  |  |  |  |  |  |  |  |  |
| 108 | HealthLens | Web | S&F TD | PT, PR | **✓** | **✓** |  |  |  |  |  |  |  |  |  |  |  |  |  |

^*^PT = Patient user type, PR = Provider user type
